# Epidemiology of porcine deltacoronavirus among Chinese pig populations in China: systematic review and meta-analysis

**DOI:** 10.3389/fvets.2023.1198593

**Published:** 2023-07-07

**Authors:** Junying Sun, Qin Zhang, Chunhong Zhang, Zhicheng Liu, Jianfeng Zhang

**Affiliations:** ^1^Institute of Animal Health, Guangdong Academy of Agricultural Sciences, Guangzhou, China; ^2^Key Laboratory of Livestock Disease Prevention of Guangdong Province, Guangzhou, China; ^3^Scientific Observation and Experiment Station of Veterinary Drugs and Diagnostic Techniques of Guangdong Province, Ministry of Agriculture and Rural Affairs, Guangzhou, China; ^4^South China Agricultural University Library, Guangzhou, China

**Keywords:** PDCoV, epidemiology, systematic review, meta-analysis, Chinese pig population

## Abstract

Porcine deltacoronavirus (PDCoV) is a newly emerging and important porcine enteropathogenic coronavirus that seriously threatens the swine industry in China and worldwide. We conducted a systematic review and meta-analysis to access the prevalence of PDCoV infection in pig population from mainland China. Electronic databases were reviewed for PDCoV infection in pig population, and meta-analysis was performed to calculate the overall estimated prevalence using random-effect models. Thirty-nine studies were included (including data from 31,015 pigs). The overall estimated prevalence of PDCoV infection in pigs in China was 12.2% [95% confidence interval (CI), 10.2–14.2%], and that in Central China was 24.5% (95%CI, 16.1–32.9%), which was higher than those in other regions. During 2014–2021, the estimated prevalence of PDCoV infection was the highest in 2015 at 20.5% (95%CI, 10.1–31.0%) and the lowest in 2021 at 4.8% (95%CI, 2.3–7.3%). The prevalence of PDCoV infection in sows was 23.6% (95%CI, 15.8–31.4%), which was higher than those in suckling piglets, nursery piglets, and finishing pigs. The prevalence of PDCoV infection was significantly associated with sampling region, sampling year, pig stage, and clinical signs (diarrhea). This study systematically evaluated the epidemiology of PDCoV infection in Chinese pig population. The findings provide us with a comprehensive understanding of PDCoV infection and are beneficial for establishing new controlling strategies worldwide.

## Introduction

Coronaviruses (CoVs) cause respiratory and gastrointestinal diseases in humans and animals. Porcine deltacoronavirus (PDCoV) is a newly emerging and important porcine enteropathogenic coronavirus that causes severe enteritis with acute diarrhea and dehydration in pigs. PDCoV infection can occur in pigs of all ages but mainly affects suckling piglets with mortality rate as high as 30–40% (1). Different from other enteric CoVs, PDCoV causes not only extensive intestinal lesions but also significant gastric lesions and mild pulmonary lesions (2). Aminopeptidase N (APN) is considered as an entry receptor of PDCoV, which is widely distributed in various tissues of multi-species, leading to presence of cross-species transmissibility (3). PDCoV can infect calves, turkeys, poultry, and mice and has independently infected children, proving its potential cross-species transmission capacity (4–6). Its spread seriously threatens the global pig industry and public health.

CoVs belong to the subfamily *Coronavirinae*, family *Coronaviridae* of the order *Nidovirales*. These positive-sense, single-stranded RNA viruses have the largest genome size among known RNA viruses. CoVs are genetically classified into four genera: Alphacoronavirus, Betacoronavirus, Gammacoronavirus, and Deltacoronavirus (DCoV) (7, 8). PDCoV belongs to the genus DCoV and has a size of approximately 25.4 kb (8, 9). Each genus of CoVs usually infects hosts in a specie-specific manner. Alphacoronavirus and Betacoronavirus infect mammals, and Gammacoronavirus primarily infect birds. DCoV can infect birds and mammals and is composed of nine avian DCoVs (*White-eye Coronavirus*; *Sparrow Coronavirus*, SpCoV; *Magpie robin Coronavirus*; *Night heron Coronavirus*; *Wigeon Coronavirus*; *Common Moorhen Coronavirus*; *Bulbul Coronavirus*; *Thrush Coronavirus*; and *Munia Coronavirus*) and three mammal DCoVs (*Asian Leopard Cats Coronavirus*, *Chinese ferretbadger Coronavirus*, and PDCoV) (9). The genome of PDCoV is similar to that of SpCoV in the same genus, indicating that the interspecific transmission of DCoV from birds to pigs may have occurred recently. The PDCoV genome organization is in the following order: 5′untranslated region (UTR), replicase open reading frame 1ab (ORF 1ab), spike (S), envelope (E), membrane (M), nucleocapsid (N), and 3′UTR, with two open reading frames encoding accessory genes nonstructural protein 6 (NS6) and nonstructural protein 7 (NS7) between M and N gene and within N gene (10–13). According to phylogenetic and comparative sequence analysis, PDCoV could be divided into four lineages: Early China, China, Thailand, USA (14). Early China and China lineages include strains from China. Thailand lineage includes strains from Laos, Vietnam and Thailand. USA lineage includes strains from USA, Mexico, Peru, Japan, Korea, and China. USA and China lineages are the major genotypes globally, and Thailand and China lineages have higher intra- and inter-lineage recombination and genetic diversity than USA lineage (14–16). Most recombination breakpoints occur in the S and ORF1ab genes, and recombination in ORF1a may result in the porcine innate immune evasion. Recombination of the S gene is a common phenomenon among CoVs; the S gene of PDCoV evolves at a lower rate than porcine epidemic diarrhea virus (PEDV) in pigs (17–22).

PDCoV was first identified in Hongkong, China in 2012. The first PDCoV strain (HKU15) was detected from rectal swabs of healthy pigs by the coronavirus diversity molecule monitoring in Hongkong (9). However, its pathogenic potential was not recognized until the first PDCoV-related diarrhea epidemic was reported in Ohio, USA in February 2014 (1). Since then, many Asian countries (Korea, China, Japan, Thailand, Laos, and Vietnam) and American countries (United States, Canada, Mexico, and Peru) have reported the PDCoV epidemic, causing a widespread concern (15, 23–28). In mainland China, since first report of PDCoV in 2015, it has quickly spread over the country. A large number of studies on PDCoV infection have been conducted in China (17, 23, 29–40). Therefore, we conducted a meta-analysis to systematically assess the prevalence and distribution characteristics of PDCoV infection in China. The findings would provide us with a comprehensive understanding of PDCoV infection and are beneficial for establishing new controlling strategies worldwide.

## Materials and methods

### Search strategy and selection criteria

This meta-analysis was reported in accordance with the Preferred Reporting Items for Systematic Reviews and Meta-Analysis statement (41). A search was conducted on PubMed, Web of Knowledge, CNKI, Wanfang, and Chongqing VIP databases between January 1, 2015 and October 31, 2022 for all studies that possibly contained data for PDCoV infection in pig populations. The databases were searched using MeSH terms and variants: “PDCoV,” “epidemiology or incidence or prevalence or investigation or surveillance or rate,” and “China or Chinese.” Studies without language limitation were included.

The eligibility for inclusion of all studies identified from the database search was independently assessed and compared by two authors. All retrieved articles were manually selected based on the relevance of publication titles and abstracts to PDCoV epidemiology. The full texts of articles considered potentially relevant based on titles and abstracts were independently reviewed by two authors. Exclusion criteria were as follows: retrospective studies, repeated studies, or nonpig studies; providing final results without sample information, such as sampling time and sample size; and sample size was <60.

### Data extraction and quality assessment

We extracted the following information from each study: first author, publication year, province of the study, administrative region, positive sample size/sample size, detection method, target gene, coinfection, and study design. The data were extracted by two authors independently, who reached a consensus through a discussion on the controversial information. The quality of included studies was evaluated according to the Grading of Recommendations Assessment, Development, and Evaluation method (42). We assigned a score to each publication. Study was awarded 1 point each when the research objective was defined, the detection method was described, the sampling method was described, subjects were classified into different subgroups, and the risk factors were determined. The publication quality was defined as low (1 point), moderate (2–3 points), or high (4–5 points). High scores indicated high quality.

### Statistical analysis

We estimated the prevalence of PDCoV infection by pooling data from included studies. We used the DerSimonian–Laird random-effect model to analyze the data (43, 44), and compared the differences using Wilcoxon two-sample test or t-test. A forest plot was used to present combined estimates with 95% CIs.

We evaluated statistical heterogeneity using *p* and *I*^2^ statistics, and it was considered insignificant only when *p* > 0.1 and *I*^2^ < 50%. The fixed-effect model was adopted in the absence of publication heterogeneity; otherwise, the random-effect model was used. Potential publication bias was assessed via a funnel plot, Egger’s regression test, and Begg’s test. Sensitivity analysis was conducted by modifying the inclusion criteria of this meta-analysis. The investigated factors were sampling region, sampling year, and pig stage. All the analysis was conducted using the Stata software (version 12.0, Stata Corporation, College Station, Texas).

## Results

### Literature search

**Figure 1:**
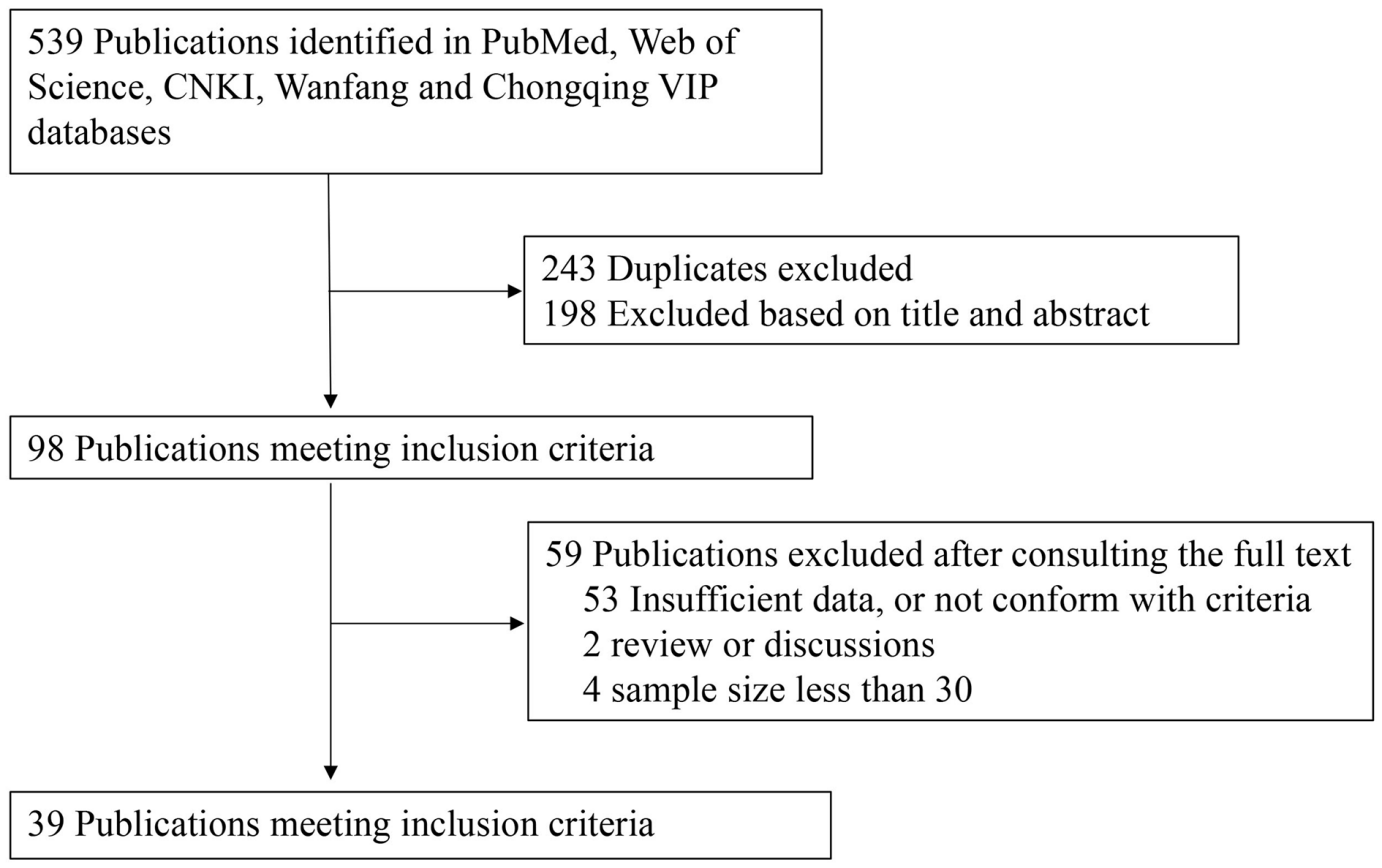
Data search and selection.

As shown in Figure 1, the literature search yielded 539 relevant studies (226 studies in English and 313 studies in Chinese), of which 243 were duplicates. After the title and abstract of each article were carefully reviewed, 98 articles were considered potentially valuable, and their full texts were retrieved for detailed evaluation. After the full text was reviewed, 59 potentially relevant articles were excluded from this meta-analysis. Among them, 53 articles did not provide required sufficient data or did not meet the inclusion criteria; 4 articles had a sample size of <60; and two articles were review papers. Finally, 39 publications were included for our meta-analysis.

### Characteristics of included studies

The characteristics of the included studies are listed in Table 1. The articles were published between January 1, 2015 and October 31, 2022 and covered 25 provinces in China. A total of 31,015 pig samples and 3,149 PDCoV-positive cases were included in the meta-analysis. In terms of epidemiological design, all 39 publications were cross-sectional studies and calculated period prevalence. Among them, 14 papers were written in English and 25 in Chinese. According to the established criteria, 25 publications were of high quality (4 or 5 points) and 14 publications were of moderate quality (2 or 3 points).

**Table 1:** Characteristics of the included studies for PDCoV infection among pigs in China.

Reference	Province	Region	No. positive/examined	Coinfection/rate	Detection Method/Target Gene	Study design	Quality score
Dong et al. (29)	Anhui, Jiangsu, Hubei, Guangxi	East China/South China/Central China	14/215	PEDV/TGEV (50%)	RT-PCR/M/N gene	C-S	5
Song et al. (23)	Jiangxi	Central China	120/356	PEDV (58.3%)	RT-PCR/N gene	C-S	5
Su et al. (30)	Heilongjiang	Northeast China	30/109	-	ELISA/N protein	C-S	3
Mai et al. (31)	Guangdong	South China	55/252	PEDV/TGEV/RoRV/PKV (49.1)	RT-PCR/M gene	C-S	5
Zhai，et al. (32)	Guangdong, Hainan, Guangxi	South China	5/390	PEDV (100%)	RT-PCR/N gene	C-S	5
Luo et al. (33)	Hebei	North China	96/871	-	ELISA/M protein	C-S	4
Wang et al. (34)	Gansu, Qinghai, Sichuan	Northwest China/ Southwest China	7/189	PEDV (42.9%)	RT-PCR/M gene	C-S	4
Jia et al. (35)	Heilongjiang, Jilin, Liaoning	Northeast China	26/672	-	rRT-PCR/N gene	C-S	3
Zhang et al. (36)	5 provinces	South China/ East China/ Central China	813/2987	PEDV/TGEV /SADS-Cov/ PoRV (90.1%)	RT-PCR/N gene	C-S	5
Zhang et al. (17)	18 provinces	7 districts	94/719	PEDV (36.2%)	rRT-PCR/M gene	C-S	3
Zhang et al. (37)	Henan	Central China	101/430	PEDV/TGEV	RT-PCR/S gene	C-S	5
Feng et al. (38)	Sichuan	Southwest China	84/634	PEDV (56.0%)	RT-PCR/−	C-S	5
Shi et al. (39)	Shanghai	East China	26/753	-	RT-PCR/M gene	C-S	3
Li et al. (40)	8 provinces	4 districts	150/7107	PEDV/PoRV (16.7%)	RT-PCR/M gene	C-S	4
Ren et al. (45)	Sichuan, Chongqing	Southwest China	6/222	-	RT-PCR/−	C-S	3
Zhang et al. (46)	Jiangxi	Central China	78/249	-	RT-PCR/N gene	C-S	4
Peng et al. (47)	Sichuan	Southwest China	20/60	PEDV/PoRV (85.0%)	RT-PCR/−	C-S	3
Zhou et al. (48)	Guangdong	South China	47/273	PEDV (91.5%)	RT-PCR/−	C-S	3
Liu et al. (49)	Sichuan	Southwest China	16/226	-	RT-PCR/M gene	C-S	3
Luo et al. (50)	Hebei	North China	22/130	PEDV (13.6%)	rRT-PCR/N gene	C-S	3
Shan et al. (51)	Zhejiang	East China	12/282	-	rRT-PCR/−	C-S	3
Song et al. (52)	Guangdong	South China	56/420	PEDV (44.6%)	RT-PCR/−	C-S	4
Xu et al. (53)	Zhejiang	East China	21/546	PEDV/TGEV/PoRV (18.2%)	RT-PCR/N gene	C-S	5
Feng et al. (54)	Sichuan	Southwest China	7/141	PEDV (57.1%)	RT-PCR/N gene	C-S	5
He et al. (55)	Guangxi	South China	70/1547	PEDV/PoRV (32.9%)	RT-PCR/−	C-S	4
Hou et al. (56)	Hebei	North China	105/570	-	ELISA/S1 protein	C-S	4
Lu et al. (57)	Tianjin	North China	417/1519	PEDV/TGEV/PoRV (−)	RT-PCR/M gene	C-S	4
Duan et al. (58)	Guangxi	South China	76/914	PEDV/PoRV (26.3%)	RT-PCR/−	C-S	4
Feng et al. (59)	Sichuan	Southwest China	51/430	-	ELISA/M protein	C-S	4
Ma et al. (60)	Shanghai	East China	25/518	PEDV/PKV/PAstV (96%)	RT-PCR/M gene	C-S	3
Shi et al. (61)	Guangxi	South China	46/792	PEDV/TGEV/PoRV (54.3%)	RT-PCR/N gene	C-S	3
Yan et al. (62)	Guangxi	South China	83/1463	PEDV/TGEV/PoRV (79.5%)	RT-PCR/N gene	C-S	4
Chang et al. (63)	5 provinces	East China	166/594	PEDV/TGEV/PoRV (58.3%)	RT-PCR/N gene	C-S	5
Li et al. (64)	Henan	Central China	54/154	PEDV (68.5%)	RT-PCR/M gene	C-S	3
Duan et al. (65)	Guangxi	South China	92/1206	PEDV/PoRV (13.6%)	rRT-PCR/−	C-S	5
Li et al. (66)	Xinjiang	Northwest China	11/1388	PEDV (36.4%)	RT-PCR/S gene	C-S	5
Zhu et al. (67)	Shanxi	Northwest China	12/184	-	RT-PCR/N gene	C-S	3
Wang et al. (68)	Hunan	Central China	0/303	PEDV/TGEV/PoRV (−)	rRT-PCR/−	C-S	5
Wang et al. (69)	Xinjiang	Northwest China	35/1200	PEDV/TGEV/PoRV (97.1%)	RT-PCR/−	C-S	4

C-S, cross-sectional study; rRT-PCR, real-time reverse transcription-polymerase chain reaction; ELISA, enzyme-linked immunosorbent assay; PEDV, porcine epidemic diarrhea virus; TGEV, transmissible gastroenteritis virus; PoRV, porcine rotavirus; PKV, porcine kobuvirus; PAstV, porcine astrovirus; SADS-CoV, swine acute diarrhea syndrome coronavirus.

### Prevalence of PDCoV infection in administrative regions of China

**Figure 2:**
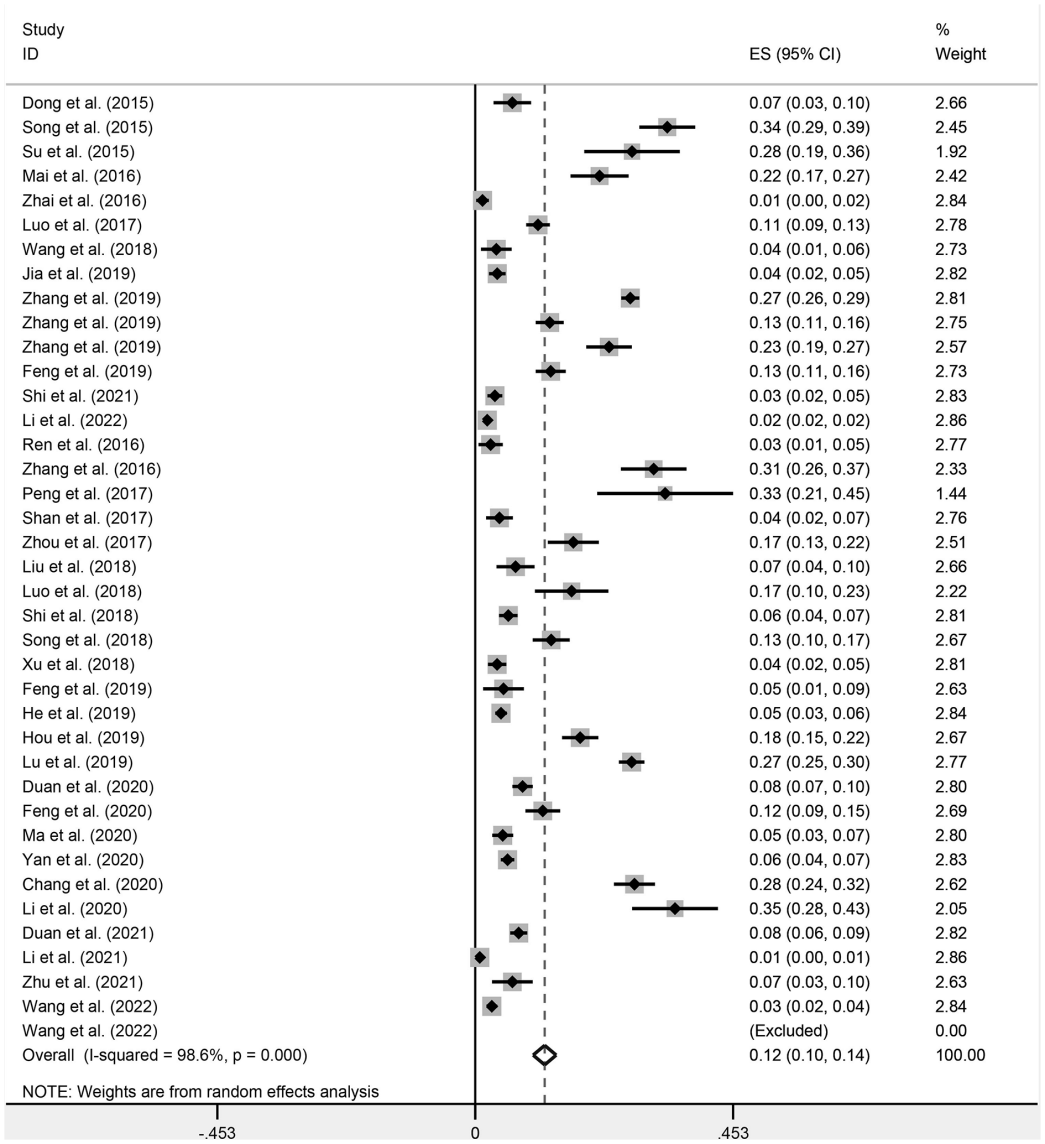
Meta-analysis of PDCoV infection among pigs in China with random-effect analysis.

**Figure 3:**
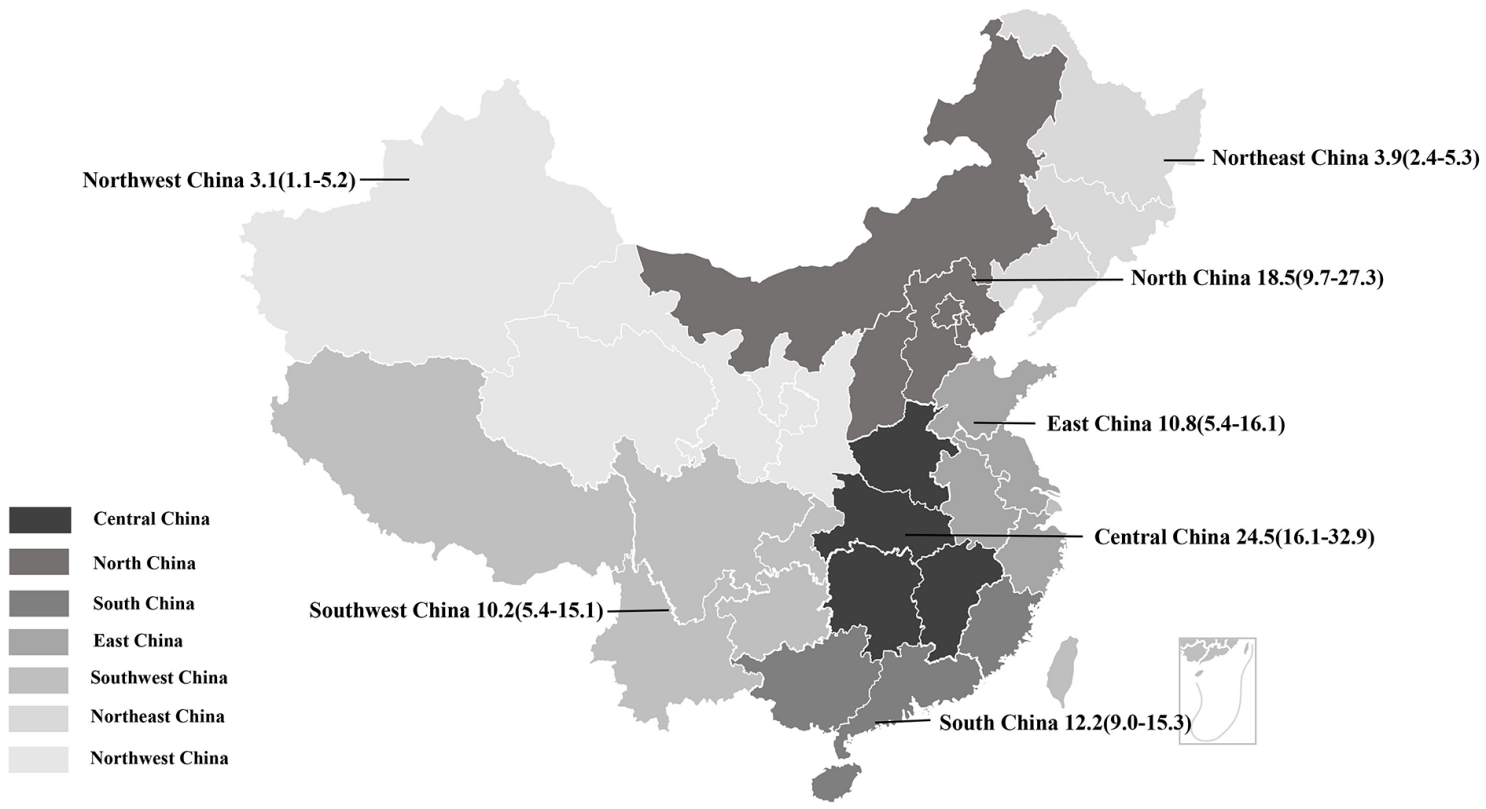
Geographical distribution of PDCoV infection among pigs in China. Pooled prevalence rate (%) and 95%CI are shown for each district.

The estimated pooled prevalence of PDCoV infection in pig population from mainland China was 12.2% (95%CI, 10.2–14.2%; Table 1; Figure 2). The prevalence rates of PDCoV infection in Central China, North China, and South China were 24.5% (95%CI, 16.1–32.9%), 18.5% (95%CI, 9.7–27.3%), and 12.2% (95%CI, 9.0–15.3%), respectively. These rates were higher than those in other administrative regions (Figure 3; Table 2). By contrast, the PDCoV positive rates in Northeast China and Northwest China regions were low with percentages of 3.9% (95% CI, 2.4–5.3%) and 3.1% (95% CI, 1.1–5.2%), respectively (Figure 3). Among the 39 studies, 28 reported coinfections. Coinfection diarrhea viruses included PEDV, *transmissible gastroenteritis virus* (TGEV), *porcine rotavirus* (PoRV), *porcine kobuvirus*, *swine acute diarrhea syndrome coronavirus*, and *porcine astrovirus*; the coinfection rate accounted for 13.6–100% of the PDCoV infection rate (Table 1).

**Table 2:** Results of the subgroup analyses on PDCoV infection among pigs in China.

category	Subgroup	No. studies	No. examined	No. positive	Prevalence (%) (95%CI)	Heterogeneity		
						*I*^2^(%)	*p*-value	Х^2^
Region	Central China	5	3,703	991	24.5 (16.1–32.9)	95.6	0.000	90.02
	North China	4	3,090	640	18.5 (9.7–27.3)	97. 3	0.000	111.03
	South China	11	7,773	691	12.2 (9.0–15.3)	96.9	0.000	322.64
	East China	6	2,774	272	10.8 (5.4–16.1)	97.3	0.000	182.69
	Southwest China	6	1713	184	10.2 (5.4–15.1)	92.3	0.000	65.28
	Northeast China	1	672	26	3.9 (2.4–5.3)	-	-	0.00
	Northwest China	4	2,916	65	3.1 (1.1–5.2)	89.2	0.000	27.79
Sampling year	Before 2014	5	2008	411	15.6 (4.5–26.8)	98.3	0.000	237.32
	2015	4	1,269	253	20.5 (10.1–31.0)	94.8	0.000	57.69
	2016	9	2,490	454	18.2 (12.3–24.0)	93.9	0.000	130.54
	2017	13	4,084	621	12.7 (8.0–17.4%)	97.4	0.000	469.30
	2018	13	6,385	559	11.3 (7.6–14.9)	97.7	0.000	523.71
	2019	6	2,648	129	8.8 (5.1–12.6)	90.2	0.000	50.92
	2020	2	2,318	46	2.0 (1.3–2.6)	2.3	0.312	1.02
	2021	3	2,677	103	4.8 (2.3–7.3)	88.5	0.000	17.34
Pig stage	Sow	9	1,685	418	23.6 (15.8–31.4)	93.1	0.000	115.42
	Suckling piglet	9	4,381	927	20.4 (11.5–29.4)	98.6	0.000	552.45
	Nursery piglet	3	857	107	10.9 (2.5–19.3)	94.1	0.000	33.83
	Finishing pig	3	422	61	14.1 (9.0–19.3)	53.4	0.117	4.29

### Subgroup analysis

All subgroup analyses included sampling region, sampling date, pig stage, and clinical signs (diarrhea). Among the seven administrative regions of China, the estimated prevalence of PDCoV infection in pigs in Central China was the highest at 24.5% (95%CI, 16.1–32.9%), and that of Northwest region was the lowest at 3.1% (95% CI, 1.1–5.2%; Table 2; Figure 3). During 2014–2021, the estimated prevalence of PDCoV infection was the highest in 2015 at 20.5% (95%CI, 10.1–31.0%) and the lowest in 2021 at 4.8% (95%CI, 2.3–7.3%), showing a downward trend (Table 2). The prevalence rates of PDCoV infection in sows and suckling piglets were 23.6% (95%CI, 15.8–31.4%) and 20.4% (95%CI, 11.5–29.4%), respectively, which were significantly higher than those in nursery piglets and finishing pigs (Table 2). The prevalence of PDCoV infection was significantly associated with sampling region, sampling date, pig stage, and clinical signs (diarrhea) but was insignificantly associated with detection method and target gene.

### Publication bias and sensitivity analysis

**Figure 4:**
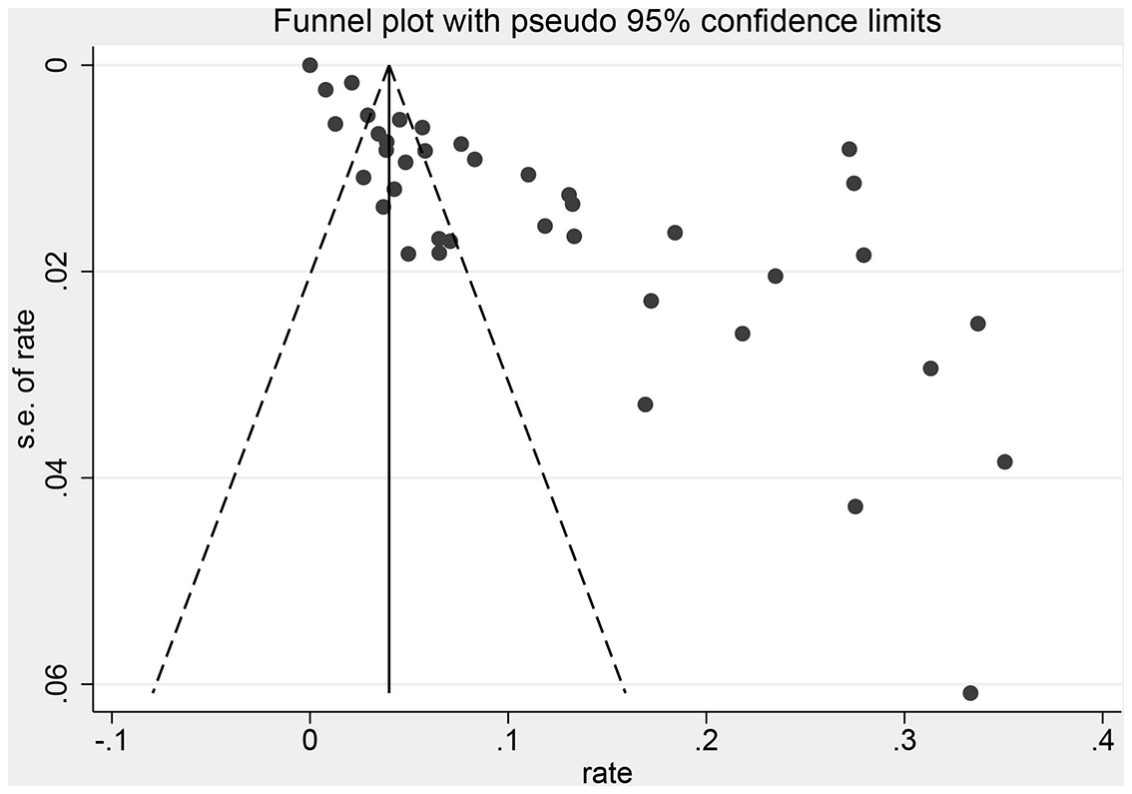
Funnel plot with pseudo 95%CIs for the examination.

The funnel forest plot was used to measure and illustrate the degree of publication bias of selected studies. The funnel plot was asymmetrical to the overall prevalence (Figure 4), suggesting significant bias in the studies selected for our analysis. A sensitivity analysis was conducted by excluding one study each time to determine whether modification of the inclusion criteria for the meta-analysis would affect the final results. All results were insignificantly changed (data not shown).

## Discussion

Coronavirus endangers human and animal health and thus causes serious public health problems and huge economic losses. PEDV and TGEV, which belong to the genus Alphacoronavirus, are two major diarrheal pathogens endangering the pig industry. Severe acute respiratory syndrome coronavirus (SARS), middle east respiratory syndrome coronavirus, and SARS-CoV-2, which belong to the genus *Betacoronaviru*s, have caused three pandemics in human history (70–72). Infectious bronchitis virus, which belongs to the genus *Gammacoronavirus*, is the main pathogen of respiratory diseases in poultry industry. DCoV is the fourth coronavirus genus formally classified by the International Committee on Taxonomy of Viruses in 2012. PDCoV diarrhea broke out in the USA for the first time in 2014, causing significant economic losses in the American swine industry, and then spread across many countries of Asia and America.

This study is the first meta-analysis and systematic review of PDCoV infection in pig herds in China. Studies on PDCoV infection in pigs from 25 provinces in China were included, all of which were cross-sectional. The pooled prevalence of PDCoV in China reached 12.2%, which indicated that PDCoV occurs extensively in Chinese pig herds. Coinfection with other enteric pathogens was common among PDCoV-positive samples. Among these pathogens, PEDV, TGEV, and PoRV had the highest frequency of coinfection. This situation implied that the current causes of diarrhea among Chinese pig populations are complex and diverse, and coinfection may cause severe clinical symptoms.

Several molecular and immunological methods have been developed to detect PDCoV. Among the molecular methods, specific RT-PCR remains the ideal choice for detection of PDCoV. Immunological methods can determine previous exposure to PDCoV and define antibody responses to infection and vaccination. Among the included papers, 35 used RT-PCR method (5 papers used rRT-PCR) and 4 utilized ELISA method. In clinical diagnostic testing, S, M, and N genes are the most commonly used diagnostic targets for PDCoV infection.

Subgroup analyzes were performed by sampling region, sampling date, and pig stage. From the perspective of geographical distribution, PDCoV was ubiquitous in pig populations in China and has large regional differences. The prevalence rates of PDCoV infection in Northeast China and Northwest China were comparatively low. On the contrary, the prevalence rates in Central China, North China, and South China were high possibly due to the large amount of pig production, high frequency of pig transport, and high humidity of climate in these regions. From the perspective of time distribution, epidemic reports have been available every year since the first report of the epidemic in mainland China in 2015. In the 1st year of the initial outbreak of the epidemic, the prevalence of PDCoV was the highest at 20.5%. Thereafter, it gradually stabilized and reached 4.8% in 2021. This situation showed that PDCoV is still prevalent in pigs in China and remains an important pathogen of porcine diarrheal disease. In terms of infected pigs, PDCoV can infect pigs of all ages. However, the clinical condition is severe in piglets. Our review found that the prevalence of PDCoV infection was significantly higher in sows (23.6%) and suckling pigs (20.4%) than in nursery (10.9%) and finishing pigs (14.1%). These results suggested that piglets are at greater infection risk, leading to high mortality from PDCoV than those of adult pigs. Moreover, the transmission of presence of virus in sows cannot be ignored.

In summary, this review reflects the trend of PDCoV infection prevalence in swine populations in China. However, this meta-analysis has certain limitations. For example, sample sizes were low in some regions (or low sample sizes were reported in certain cases). Analysis was also limited to date of sampling, geographic location, gene of interest, pig stage, and clinical signs. Other potentially influential factors, such as farm size, breed, and sampling season of pigs, were not analyzed. All data were from pigs with diarrhea. Additional samples of healthy pigs are suggested to be included to assess the infection of PDCoV in pigs in China. The abovementioned factors should be considered when conducting epidemiological studies in the future.

## Conclusion

Our meta-analysis shows a high prevalence (12.4%) of PDCoV infection in Chinese pig herds. The prevalence rate is significantly associated with sampling region, sampling year, pig stage, and clinical signs in pigs (diarrhea). Therefore, biosecurity prevention and control should be strengthened to reduce the spread of PDCoV between regions. Climate, such as humidity and temperature, correlates with the breakout of PDCoV, Thus, this study recommends to keep pig house dry and warm. Surveillance of PDCoV and detection of other diarrhea pathogens should be strengthened in suckling piglets and sows due to high morbidity in suckling piglets and high virus-carrying rate in sows. The prevalence of PDCoV shows a downward trend; however, consideration of susceptibility of coronavirus to mutation, recombination, and cross-species transmission and continuous surveillance studies in swine remain essential (including non-diarrheal swine) to monitor the geographical spread and incidence trend of PDCoV and detect the genetic evolution.

## Data availability statement

The original contributions presented in the study are included in the article/supplementary material, further inquiries can be directed to the corresponding author.

## Author contributions

JS conceptualized the paper and wrote the manuscript. JS, QZ and JZ collected and analyzed the data. CZ and ZL revised the manuscript. All authors contributed to the article and approved the submitted version.

## Funding

This work was supported by the National Key Research and Development Program of China (grant number 2022YFD1800801-02); the Science and Technology Planning Project of Guangzhou (grant numbers 202002030456 and 20212100050); the Science and Technology Planning Project of Guangdong Province (grant number 2021B1212050021).

## Conflict of interest

The authors declare that the research was conducted in the absence of any commercial or financial relationships that could be construed as a potential conflict of interest.

## Publisher’s note

All claims expressed in this article are solely those of the authors and do not necessarily represent those of their affiliated organizations, or those of the publisher, the editors and the reviewers. Any product that may be evaluated in this article, or claim that may be made by its manufacturer, is not guaranteed or endorsed by the publisher.
